# Network motif comparison rationalizes Sec1/Munc18-SNARE regulation mechanism in exocytosis

**DOI:** 10.1186/1752-0509-6-19

**Published:** 2012-03-16

**Authors:** Tian Xia, Jiansong Tong, Shailendra S Rathore, Xun Gu, Julie A Dickerson

**Affiliations:** 1 Biomedical Informatics Center, Northwestern University, Chicago, IL 60611, USA; 2 Department of Biochemistry, Biophysics and Molecular Biology, Iowa State University, Ames, IA 50011, USA; 3 Department of Molecular, Cellular, and Developmental Biology, University of Colorado at Boulder, Boulder, CO 80309, USA; 4 Department of Genetics, Development and Cell Biology, Iowa State University, Ames, IA 50011, USA; 5 Program of Bioinformatics and Computational Biology, Iowa State University, Ames, IA 50011, USA

## Abstract

**Background:**

Network motifs, recurring subnetwork patterns, provide significant insight into the biological networks which are believed to govern cellular processes.

**Methods:**

We present a comparative network motif experimental approach, which helps to explain complex biological phenomena and increases the understanding of biological functions at the molecular level by exploring evolutionary design principles of network motifs.

**Results:**

Using this framework to analyze the SM (Sec1/Munc18)-SNARE (*N*-ethylmaleimide-sensitive factor activating protein receptor) system in exocytic membrane fusion in yeast and neurons, we find that the SM-SNARE network motifs of yeast and neurons show distinct dynamical behaviors. We identify the closed binding mode of neuronal SM (Munc18-1) and SNARE (syntaxin-1) as the key factor leading to mechanistic divergence of membrane fusion systems in yeast and neurons. We also predict that it underlies the conflicting observations in SM overexpression experiments. Furthermore, hypothesis-driven lipid mixing assays validated the prediction.

**Conclusion:**

Therefore this study provides a new method to solve the discrepancies and to generalize the functional role of SM proteins.

## Background

Cellular processes are governed by complex molecular interaction networks where the molecular components and the interactions between them are represented by nodes and edges, respectively. Intensive studies of local and global organizing principles of the networks show the inherent simplicity of biological networks: modularity and reusability [1-5]. These networks can be decomposed into independent functional modules. Small recurring subnetworks that perform specific cellular subfunctions (termed network motifs) are largely reused to build the functional modules. The network motifs also show stability or robustness to environmental conditions and evolutionary dynamics and therefore are viewed as building blocks of the complex networks [6,7]. The experimental approach of network motif identification is extensively applied for modeling specific cellular processes [8].

However, whereas studies have mainly focused on modeling or analysis of topological or kinetic features of network motifs in a single cell type or species, network motifs can be used to reflect dynamical and evolutionary adaptations to meet physiological variances over a time course. Integrating the dynamics across species is particularly important in modeling cellular processes through protein interaction networks. Many of the biological processes mediated by protein interaction networks are highly evolutionarily conserved or related across species. The evolutionary dynamics of biological processes shape the network structure over large time scales. For instance, protein interaction networks are believed to evolve through genetic sequence mutation or gene duplication [9,10]. The gene duplication can create a new node which owns identical edges to the original node, but after being duplicated it could lose its functions (corresponding interaction edges are eliminated). Mutations of a gene sequence can modify the interfaces or domains of its protein product and lead to the emergence of new or loss of existing protein interaction patterns [11]. Therefore, information about evolutionary dynamics is invaluable for network modeling of biological systems.

**Figure 1:**
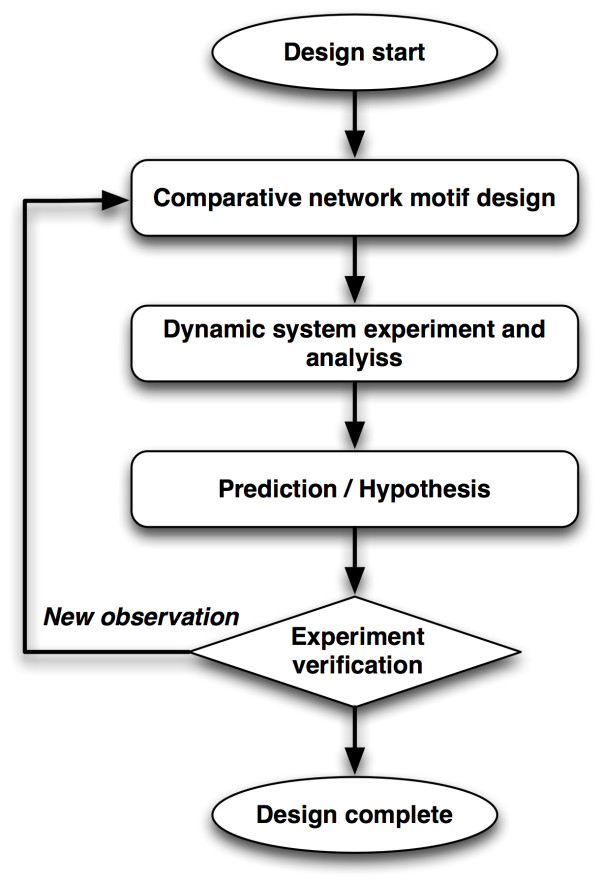
**Experimental Diagram of Comparative Network Motif Design Modeling**.

We developed an analysis framework on the basis of comparative network motif design (Figure 1). Given a network motif structure representing a specific biological function in one cell type or species, this approach utilized a comparative modeling strategy to connect it with other network motifs which are evolutionarily related to each other. By capturing the evolutionary dynamics of target biological systems, the comparative modeling framework is empowered to (i) identify the functional roles of poorly characterized proteins and interactions and (ii) further decipher the underlying regulatory mechanisms of complicated cellular processes.

We applied the framework to study SM-SNARE-mediated exocytic membrane fusion processes in yeast and neurons. As for many essential biological processes, intracellular membrane fusion is mediated by interactions among a series of evolutionarily conserved proteins. SNARE proteins are viewed as a critical component in execution of vesicle membrane fusion with the target plasma membrane, forming a helical-bundle complex termed a SNAREpin through interactions of v-SNAREs (vesicle - associated SNARE proteins) and t-SNAREs (target membrane associated SNARE proteins) [12,13]. SM (Sec/Munc-18) proteins are essential regulators responsible for controlling the formation of SNAREpin complexes by diverse binding modes with SNAREs [14,15]. These binding modes show high heterogeneity between different organisms or trafficking pathways [16]. This binding diversity brings uncertainty and complexity into the interaction network of vesicular fusion regulation and therefore poses a challenge to understanding the key functional roles of the SM protein family in exocytosis. SM proteins have been documented to be both positive and negative regulators of fusion, and studies of overexpression of SM proteins have produced conflicting observations [17-20].

Applying our modeling framework, we comparatively constructed two ensemble SM-SNARE network motifs (SSNM) in the exocytic network based on the binding mode information curated from current literature: the cascade-like SSNM in yeast and the feedback-loop-like SSNM in neuronal synaptic pathways. Comparative dynamical analysis revealed bifurcation behavior in the neuronal system which was different from hyperbolic response behaviors in the yeast system and provides a way to explain the conflicting experimental observations of SM overexpression in neuronal systems. Furthermore, the comparative topological analysis revealed that the closed binding mode of Munc18-syntaxin-1 in neuronal SSNM may be the critical factor that brings the complexity to synaptic exocytosis in terms of network topology and system behaviors compared to yeast exocytosis. Furthermore, *in silico *mutation experiments confirmed that the bifurcation behaviors resulted from the closed binding mode of Munc18-syntaxin-1. Our reconstitution lipid-mixing assay experiments based on wildtype and mutant SNARE proteins confirmed the prediction that the closed binding mode of Munc18-syntaxin-1 (one tSNARE protein) in neuronal SSNM explains d the divergence of yeast and neuronal SM-SNARE system behaviors. Therefore it reconciles s the discrepancy y in studies of over-expressed SM protein from a system regulation point of view. To test the robustness and extensibility of the model, we further expanded the neuronal SSNM with other exocytic proteins, which may regulate SM and SNARE proteins.

## Results

For comparative modeling of network motifs for the complicated molecular machinery of exocytic membrane fusion, we outlined a three-step strategy, integrating prediction-driven *in vitro *experiments with *in silico *network motif modeling. The strategy is shown in Figure 1. (i) First, the network motif design provides a rational description for key parts of the biological system of interest by decomposing a complicated network into simple regulatory network motifs that carry out specific functions. The comparative generation of network motifs enables us to infer potential protein functions by comparing targets with well-studied and evolutionarily-related proteins and systems across species. Second, the dynamical analysis and *in silico *experiments link the molecular architecture to cellular function and demonstrate system behaviors. It can identify key factors which may introduce the divergence of system behaviors and provide predictions regarding underlying regulatory mechanisms of the target system. Third, experiments are designed to verify the predictions and new components are included to test the robustness and extensibility of the model.

### Comparison of network motif models reveals that the closed binding mode of neuronal munc18-syntaxin underlies the complexity in neuronal membrane fusion

#### Comparative design of SM-SNARE network motifs

**Figure 2:**
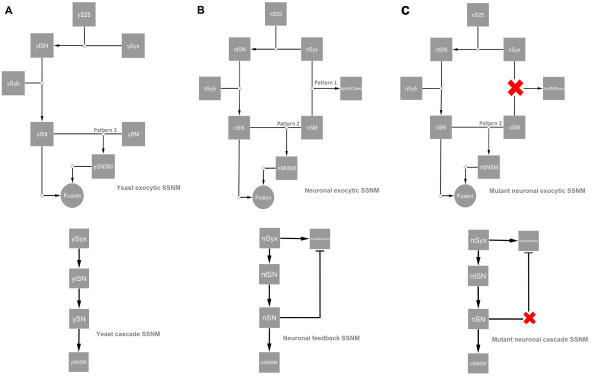
**SM-SNARE Network motif design**. (a) Formulated diagram of the yeast SSNM in exocytosis. ySyx: yS25, ySyb and ySM describe Sso1p(yeast syntaxin), Sec9p(yeast SNAP25), Snc1/2p(yeast synaptobrevin) and Sec1p(SM), respectively. The logic network diagram on the left shows the cascade-like yeast SSNM. (b) Formulated diagram of neuronal network in synaptic exocytosis, nSyx, nS25, nSyb and nSM describe syntaxin-1, SNAP25, VAMP(neuronal synaptobrevin) and Munc18-1(neuronal SM), respectively. The logic network diagram on the left shows the feedback structure with modulation in the neuronal synaptic system. (c) Formulated diagram of mutant neuronal network in synaptic exocytosis, nSyx, nS25, nSyb and nSM describe syntaxin-1, SNAP25, VAMP (neuronal synaptobrevin) and Munc18-1(neuronal SM), respectively. The logic network diagram on the left shows that the feedback structure is blocked due to mutant nSyx. These network motifs are designed by CytoModeler based on the Cytoscape platform.

We first constructed SM-SNARE network motifs because SM proteins and SNAREs play central positions in the protein interaction network of intracellular membrane fusion. Two SM-SNARE network motifs are reconstructed for yeast and neuronal synaptic exocytosis because they represent two fundamental types of exocytosis: constitutive and regulated (Figure 2). We developed CytoModeler software based on Cytoscape platform [21] to facilitate the network motif design and experiment. Please see Additional file 1 for details. In Figure 2, the reaction arrows represent reversible reactions and the rate constants can be input in the software interface. In Figure 2, the nodes represent the SNARE, SM, and reactant complex. The edges describe the interactions between them. The process of exocytic fusion is formalized as a feed forward set of interactions between the SNARE proteins and the SM protein. (To simplify the notation and emphasize homology between the networks, Additional file 1: Table S1 gives the naming conventions for all models). The design is based on binding modes between the two protein families. In general, these binding modes can be categorized according to the binding protein partners of SM: the mono-SNARE (pattern 1) and the SNAREpin complex (pattern 2) [15,16,22,23]. Categorizing the relationships between SNAREs and SM proteins provides a way to simplify reaction relationships between SM, SNAREs and SNARE complexes because there exist controversies and complexities about specific binding domains or peptides involved in various SM and SNARE interactions. For example, Burkhardt, et al. 2008 showed that the binary Munc18-1 and syntaxin-1 interaction involves both N-peptides and the closed conformation of syntaxin-1 and the Munc18-1-bound syntaxin-1 is only able to form a SNAREpin when the N-peptide is released from Munc18-1. However, this is contrary to findings which demonstrate that the syntaxin-1 N-peptide "stays bound to Munc18-1 when remainder of the molecule assemble into a SNARE complex" [24] and "the SNARE four-helix bundle and syntaxin N-peptide constitute a minimal complement for Munc18-1 activation of fusion" [25,26]. In Xu et al., Munc18-1 binding to VAMP2 was observed mostly during the status transition from the *trans *to *cis *SNAREpin complex [27].

In yeast, the exocytosis pathway operates continually supplying vesicles containing lipids and proteins for the plasma membrane. Yeast exocytic SNAREs Sso1p (yeast syntaxin/t-SNARE), Sec9p (yeast SNAP25/t-SNARE) and Snc1/2p (yeast synaptobrevin/v-SNARE) mediate the vesicular fusion process. Sso1p and Sec9p preassemble into the t-SNARE complex. Then, Snc1/2p associates with the complex to form the SNAREpin complex, which acts as an engine to release biochemical energy to drive the vesicular and plasma membranes together. The yeast SM protein, Sec1p, regulates the SNARE complexes and the fusion rate by directly binding to the assembled SNAREpin (pattern 2) [28,29].

In neurons, the synaptic exocytosis pathway is highly regulated in time and space, and it controls specialized neuron communication and the release of neurotransmitters contained by synaptic vesicles in response to calcium signals. Despite the regulation, the core molecular machinery of the synaptic exocytosis pathway is evolutionarily related to that of yeast. For example, neuronal t-SNAREs, syntaxin-1 and SNAP25 pre-assemble into a t-SNARE complex. The complex later reacts with VAMP (synaptobrevin/vesicle associated membrane protein) to form an assembled SNARE complex/SNAREpin. The neuronal SM protein Munc18-1 also binds to the assembled SNAREpin (pattern 2) to facilitate membrane fusion. Munc18-1 has an extra binding mode (closed mode of binding of Munc18-syntaxin) with syntaxin-1 (pattern 1), which stabilizes syntaxin-1 in the closed conformation, blocking the formation of the SNAREpin complex [14]. Furthermore, recent studies revealed that Munc18-1 was also able to interact with SNAREs or SNAREpin complex through the N-peptide of syntaxin-1. However, there are inconsistent observations regarding the mode of binding between Munc18-1 and syntaxin-1 as we discussed. Therefore, according to binding protein partners of Munc18-1, these suggested binding modes can be categorized into pattern 1 and pattern 2 respectively, while there are controversies whether the N-peptide binding of Munc18-1 and syntaxin-1 exists in the binary Munc18-1/syntaxin-1 complex or Munc18/SNAREpin complex. According to the SM-SNARE network motifs, we built dynamical models for each networks motif, enabling examination of the behavior of the system (please refer to Additional file 1).

#### Comparative in silico experiments reveal differential system behaviors of SM regulation

We investigated system behavior in response to SM regulation both in yeast and neurons, using the system models described above.

**Figure 3:**
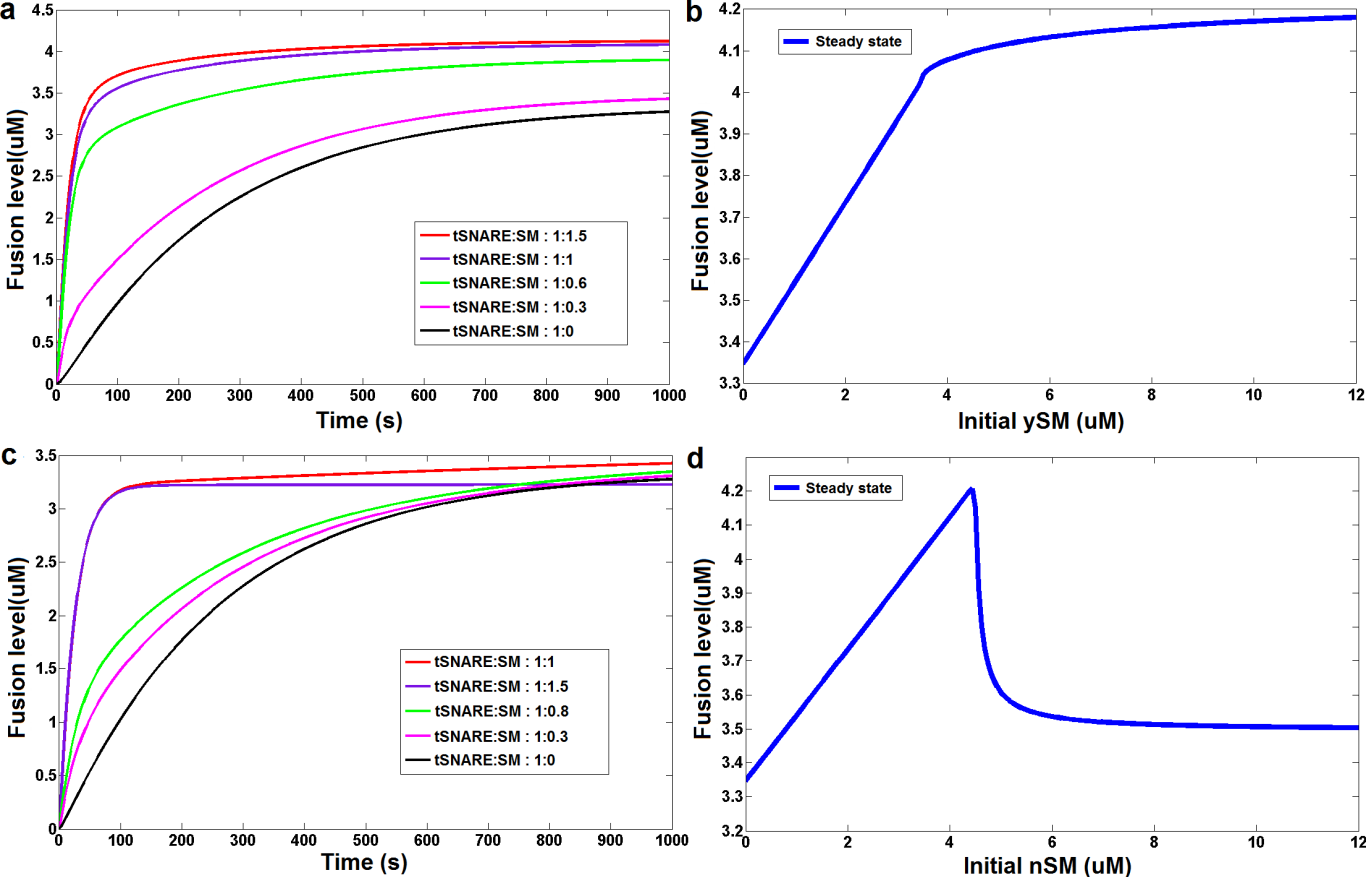
**Comparative *in silico *experiments and analyses of yeast and neuronal SM-SNARE network motifs**. (a) Fusion curves of five in silico experiments for different initial concentrations of the ySM protein using the network from Figure 2a. (b) Final fusion levels of the yeast SSNM at steady state with respect to concentration of the ySM protein. (c) Fusion curves of five in silico experiments for different initial concentrations of the nSM protein using the network from Figure 2b. (d) Final fusion levels of the neuronal SSNM at steady state with respect to the concentration of the nSM protein.

The first simulation models system behaviors of the cascade-like yeast SM-SNARE network motif with respect to the ySM protein concentration and the results show yeast SM stimulates fusion. The ySM positively regulates the fusion process as the amount of fusion shows a hyperbolic response to the ySM protein concentration. Figure 3a presents fusion curves of five simulated experiments with different ySM concentrations. Figure 3b depicts the steady-state fusion level of the system with respect to varying the initial concentration of ySM protein. This demonstrates that ySM plays a positive role that stimulates membrane fusion in both rate and amount. The simulation analysis agrees with experimental observations in lipid mixing assays [29]. In that study, recombinant yeast SM protein Sec1p was added to the yeast SNARE reconstitution liposome system at different concentrations. The effects of the stimulation on fusion show dependency on Sec1p concentration (please refer to the Figure 6D of Scott BL et al. [29]). When the levels of Sec1p are increased in the assay, a monotonic increase in fusion is observed [29]. Therefore, the experimental observations verify the yeast SSNM model prediction.

The neuronal SSNM model allows computational exploration of the system behaviors in the feedback-loop-like neuronal SM-SNARE network motif with respect to the nSM protein concentration and the results show that SM stimulates fusion in neurons but in a more complex ways than in yeast. A bifurcation behavior is observed in the neuronal SSNM model where nSM can play either a positive or negative role depending on the dose (Figure 3c and 3d): at reasonable physiological levels (the concentration of nSM is less than nSyx [16,17]) nSM effectively stimulated the fusion. However, under extreme conditions where concentration of nSM is larger than nSyx, nSM concentration shows a negative relationship with fusion efficiency. This response requires the level of nSM protein concentration to be much larger than that of t-SNARE, which is hard to achieve under normal physiological conditions *in vivo *because of the fact that syntaxin-1(tSNARE) outnumbers Munc18-1(nSM)[16,17].

### Network comparison analysis extracts the critical distinction between two SM-SNARE networks

To extract the critical factor which underlies the divergence and complexity in the yeast and neuronal exocytic systems, we next investigated the two SM-SNARE network motifs from yeast and neurons. Network comparison analysis explores the differences with respect to network structure, since the topological diversity of biological networks usually reflects the diversities of function, evolutionary selection, and regulation mechanism of cellular processes [2,6,9]. The analysis showed that the neuronal SM-SNARE binding mode (closed binding mode of Munc18-syntaxin) might be a critical factor in the structural divergence of the SM-SNARE network motifs in yeast and neurons. In the yeast SSNM, every component piece of SNAREpin/SM is sequentially assembled to an intermediate protein complex through a series of discrete levels. Therefore the network motif is cascade-like (Figure 2a). In the neuronal SSNM, there is a cascade branch similar to that in yeast. However, there is an additional branch which is introduced by the neuronal closed mode of Munc18-syntaxin binding. Due to this extra branch, nSyx (syntaxin-1) is inhibited by nSM (Munc18-1) or it plays another functional role in its interaction with nSM (Munc18-1), for example in vesicle docking [19]. These two branches actually form a feedback loop because the t-SNARE complex and SNAREpin which form through the cascade branch can also interact with nSM forming the SNAREpin/SM complex. This sequesters nSM (Munc18-1) away from nSyx (syntaxin-1) and prevents nSyx from being inhibited in the closed mode (Figure 2b).

The neuronal SM-SNARE binding mode (closed mode of binding of Munc18-syntaxin) radically changes the topology of the SM-SNARE network in neurons compared with that in yeast, even as it conserves the cascade-like branch. This predictively suggests that the binding mode drives the divergence of the SM-SNARE network motif regulation in the secretory pathways in the different systems, and introduces the complexity into the neuronal system.

### Simulated mutation confirms the critical factor in neuronal SM-SNARE network motif

**Figure 4:**
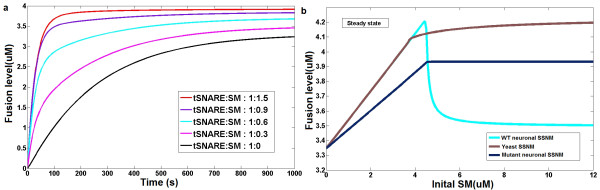
***In silico *mutant experiments and analysis of neuronal SM-SNARE network motif**. (a) Fusion curves of five in silico experiments with different initial concentrations of the nSM protein in the mutant neuronal SSNM system which eliminates the nSM(Munc18-1) binding to closed nSyx(syntaxin-1) (Figure 2c). (b) A comparison of fusion levels between the yeast SSNM, neuronal SSNM and mutant SSNM at steady state with respect to the SM protein concentration.

To test this prediction, we next performed a perturbation experiment *in silico *to eliminate the neuronal nSM binding to closed nSyx (closed mode of binding of Munc18-syntaxin) (see Figure 2c). The simulation results (Figure 4) showed that the mutant neuronal SSNM had similar behavior to the yeast SSNM: The fusion was amplified by increasing the concentration of nSM in the mutant nSM-SNARE system and the stimulation effect monotonically increased with the concentration of the mutant nSM protein. Therefore the *in silico *mutant experiment confirmed the prediction that neuronal nSM binding to closed nSyx (closed binding mode of Munc18-syntaxin) may be the key factor in inducing the structural divergence of SM-SNARE network motif in yeast and neurons.

### Prediction-driven lipid mixing assay confirms the critical factor in neuronal SM-SNARE network motif and provides explanation of regulatory mechanism to resolve conflicts observed in SM overexpression studies

To further test the predictions by our model, we utilized fluorescence resonance energy transfer-based lipid fusion assays, in which neuronal SNAREs are reconstituted into liposomes at physiologically relevant surface densities and when fusion occurs between the fluorescent donor and unlabeled acceptor vesicles the fluorescent intensity can reflect the dynamics of lipid fusion. More importantly, the reconstitution lipid mixing assay allows us to investigate the fusion event by precisely controlling the concentration ratio of SNARE proteins or other regulatory proteins.

**Figure 5:**
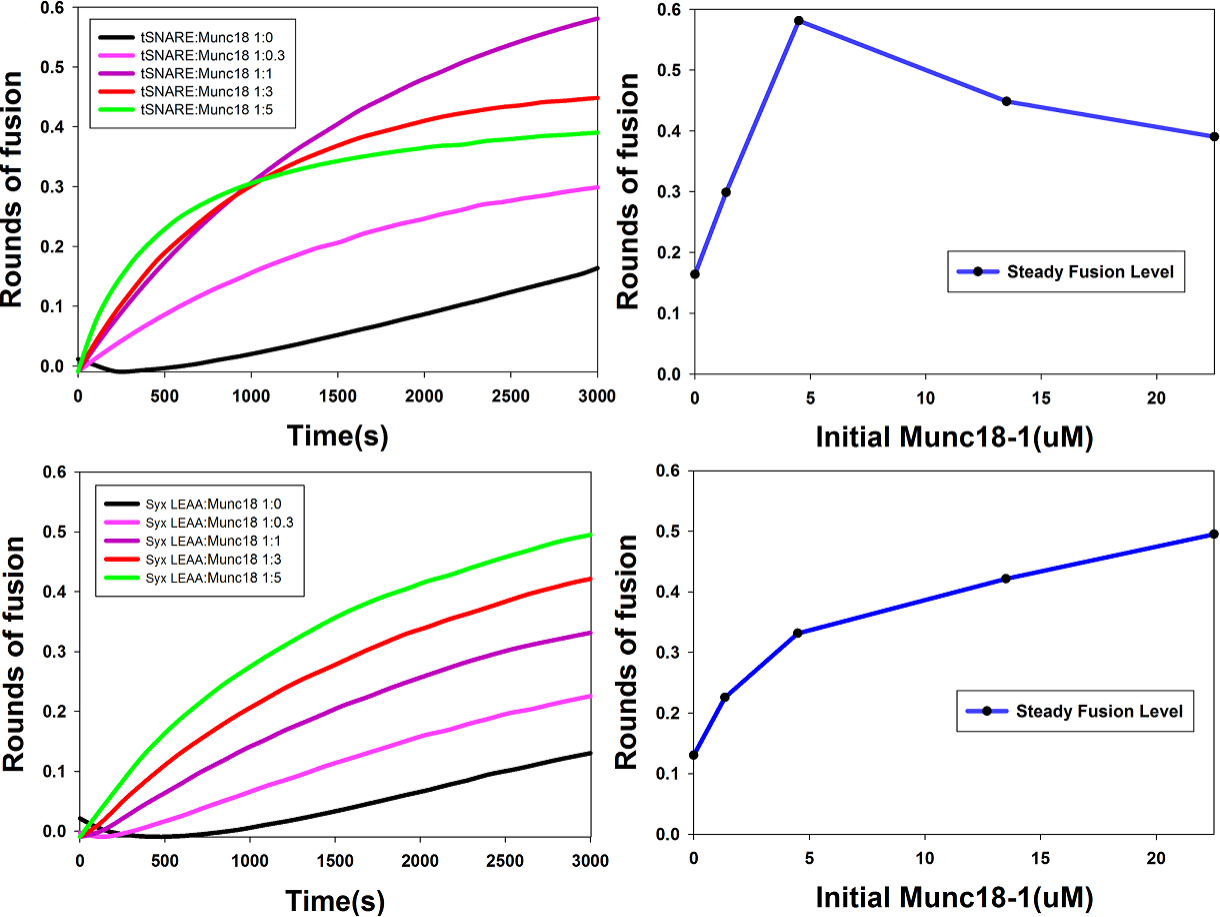
**Prediction-driven *in vitro *experiments and analysis of neuronal SM-SNARE network motif**. (a) and (b) lipid mixing assay of wildtype neuronal SM-SNARE system with different initial concentration configurations of Munc18-1. The result confirms the bifurcation behavior predicted by simulation experiments (shown in Figure 3) that when the concentration of Munc18-1 is equal to the concentration of Syntaxin-1 the fusion effect reaches maximum (purple line). (c) and (d) Mutant lipid mixing assay of neuronal SM-SNARE system with separately expressed SNAP2 and mutant syntaxin-1. The result of b and c confirmed that the complicated bifurcation behavior is introduced by the unique binding mode between Munc18-1 and Syntaxin-1 because the mutant system which deletes the binding mode shows a similar behavior to the yeast system without bifurcation phenomenon.

To test whether there is bifurcation behavior in neuronal SM-SNARE network motif, we firstly employed the lipid mixing assay for the wildtype SNARE-SM system. The tSNAREs syntaxin-1 and SNAP25 were separately expressed. The dynamics of fusion showed the exact bifurcation behavior with respect to Munc18-1(nSM) initial concentration which we observed in the network motif modeling (See Figure 5a and 5b). This not only confirms the prediction but also provides a way to explain contradictory observations of the effect of overexpression of SM protein in various cell types [18,20,30,31].

To test whether the complexity of neuronal SM-SNARE network motif is introduced by the closed binding mode of Munc18-1(nSM)-syntaxin-1, we employed the lipid mixing assay in a mutant SNARE-SM system as previously reported [26], where point mutations were introduced into syntaxin-1 (L165A and E166A). They are believed to create a constitutively "open" syntaxin-1 and therefore significantly reduce the affinity of the closed binding interaction. To examine the mutant system thoroughly, we designed experiments where SNAP25 and mutant syntaxin-1 were separately expressed. The results of the lipid mixing experiment showed that the dynamics of the fusion reaction responded to the initial concentration of Munc18-1(nSM) in a simple hyperbolic manner consistent with the prediction made by the *in silico *mutant experiment rather than a bifurcation as seen in the wild type neuronal SM-SNARE system (Figure 5c and 5d).

### Solving conflictions observed in SM overexpression experiments

**Figure 6:**
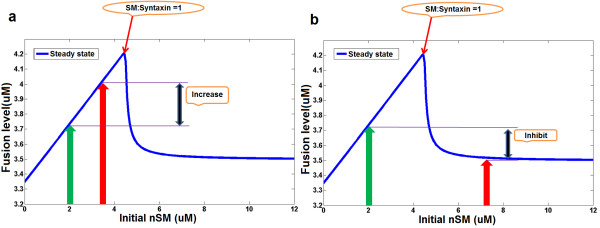
**Explaining conflictions in SM overexpression experiments**. (a) shows a scenario where the overexpression of SM may cause increased fusion. (b) shows a scenario where the overexpression of SM may cause decreased fusion. The bifurcation behavior of the neuronal SM-SNARE network motif provides an explanation for conflicting observations in SM protein overexpression experiments.

The confirmed bifurcation behavior of the neuronal SM-SNARE system provides a mechanistic explanation for the discrepancies observed in SM overexpression experiments. The overexpression of SM protein inhibited synaptic exocytosis in flies but increased exocytosis in chromaffin cells, PC12 cells and motor neurons, and had no effect on exocytosis in some studies [18,30-33]. From our analysis, we can predict that the inconsistencies might result from the bifurcation mechanism. We assume that the initial concentration of SM is less than t-SNARE (green arrow line in Figure 6). Since the dosage extent (red arrow line in Figure 6) of the overexpression of SM varies in different cell types, the outcome of systems behaviors (fusion level) shows uncertainties: when the overexpression of nSM increases the concentration of nSM beyond the bifurcation point, it results in negative regulation of fusion or no obvious effects (Figure 6b); when the overexpression of nSM maintains the concentration below the bifurcation point, it stimulates the fusion process (Figure 6a).

### Expanding the SM-SNARE network motif

In addition to SM and SNARE, many other important regulatory proteins are involved in exocytic membrane fusion, especially in neurons, such as Munc13-1, complexin, and synaptotagmin [34]. These proteins interact to form an intricate protein interaction network at a large scale. Using our framework, we can extend the model and integrate other regulatory factors in the exocytic system since it is evident that the network motif can function independently. Hierarchical combinations of the network module forms more complex biological functions, and the network module shows simplicity and robustness with a limited number of network topologies [2,6,7,35]. For instance, it is believed that Munc13 and Tomosyn are able to interact with the Munc18/syntaxin binary complex, displacing Munc18 from syntaxin [16,36,37]. Based on these observations, we expanded our network motif model by integrating the displacement factor (DF). However, the new element does not change the feedback-loop like topological structure of the original neuronal SM-SNARE network motif. Therefore, according to the motif theory, the new network motif is expected to have a similar behavior to the neuronal SSNM we discussed before. Steady state analysis of the new model confirmed the similarity as a bifurcation behavior was observed (See Additional file 1: Figure S2 in Additional file 1), showing the functional robustness of the SM-SNARE network motif.

## Discussion

This work developed a comparative strategy to facilitate network motif modeling for complex biological processes. Applying the method to SM-SNARE systems in exocytic membrane fusion, we connect the regulation mechanism of SM-SNARE to the network motif structure of the protein interaction and to the evolutionary dynamics of the network motifs. This comparative analysis indicated that the topological shift of the network motifs from yeast to neuron is a force underlying the complicated behavior of the neuron system. The prediction-driven lipid mixing assays were then designed in wildtype and mutant neuronal systems to test the findings produced by the comparative system modeling. The result further confirmed the bifurcation behavior in neuronal systems. Specifically, the bifurcation behavior of the neuronal system in response to different SM concentrations provides a new perspective on discrepancies observed in SM overexpression experiments.

This analysis also showed that the closed mode of binding of Munc18-1 to syntaxin-1 is a potentially critical contributor to divergence of network motif structure topology between yeast and neurons in exocytic membrane fusion. This binding mode is not observed in yeast exocytic membrane fusion but was recently discovered in endosomal trafficking in yeast [38]. Recent studies show that the Munc18-1/syntaxin-1 binary complex positively functions in the docking of vesicles to their target membrane, while Munc18-1 was first characterized as a negative factor in neurons because Munc18-1 reacts with syntaxin-1 in the closed conformation and therefore inhibits the syntaxin from forming a SNAREpin complex. The comparative modeling analysis *in silico *can explore the dynamical behaviors and controlling mechanism of the systems and infer potential functional roles of system elements such as SM proteins under different conditions. The prediction-driven comparative wet experiments in the trafficking systems can then be specifically designed under different conditions to test the conclusions and therefore offer a mechanistic understanding for the complex biological systems in an effective manner. Many other important regulatory proteins are involved in exocytic membrane fusion. Deciphering this complex network remains challenging, however our comparative network motif modeling offers an extensible and robust experimental framework to understand the dynamics of large-scale network in terms of elementary network patterns.

## Methods

### Protein expression and purification

Plasmid construction, mutagenesis, protein expression and purification for neuronal SNAREs have been described elsewhere [39]. Briefly, full length DNA of vesicle-associated (v-) SNARE synaptobrevin (also called VAMP2, amino acids 1-116) and soluble protein SNAP25 (amino acids 1-206) were constructed into pGEX vector as N-terminal glutathione S-transferase fusion proteins. Wild type and mutant target membrane (t-) SNARE syntaxin (amino acids 1-288) and regulator protein Munc18 were constructed into pET21 vector as the C-terminal his-tag protein. Recombinant proteins were expressed in Escherichia coli Rosetta (DE3) pLysS (Novagene). Synaptobrevin and SNAP25 were purified by affinity chromatography using glutathione-agarose beads (Sigma) by cleaving with thrombin in cleavage buffer (50 mM Tris-HCl, 150 mM NaCl, pH 8.0) for 1 hour at room temperature. Syntaxin and Munc18 were purified by his-tag nickel beads. We added 1% OG (n-octyl-β-D-glucoside) to all the proteins during purification.

### Membrane reconstitution

The procedure was described elsewhere [40]. Briefly, full length syntaxin and SNAP-25 were mixed as 1:1 ratio for 1 h under room temperature to allow for the formation of t-SNARE complex. The preformed t-SNARE complex was reconstituted with 50 mM liposomes (with size of 100 nm) containing 1-palmitoyl-2-dioleoyl-*sn*-glycerol-3-phosphatidylcholine (POPC) and 1, 2-dioleoyl-*sn*-glycerol-3-phosphatidylserine (DOPS) (molar ratio 65:35) with a lipid/protein ratio of 100:1. The v-SNARE synaptobrevin was reconstituted with another 10 mM liposome containing POPC, DOPS, NBD-PS (1, 2-dioleoyl-*sn*-glycerol-3-phosphoserine-*N*-(7-nitro-2-1, 3-benzoxadiazol-4-yl)) and rhodamine-PE (1, 2-dioleoyl-*sn*-glycerol-3-phosphoethanolamine-*N*-(lissamine rhodamine B sulfonyl)) (molar ratio 62:35:1.5:1.5) with the lipid/protein ratio of 100:1. Two reconstituted liposomes were dialyzed overnight using dialysis buffer (25 mM Hepes, 100 mM KCl, 10% glycerol, pH 7.4) to remove detergent OG. After dialysis, the solution was centrifuged at 10000 x g to remove protein and lipid aggregates.

### Lipid mixing assay

To measure the lipid mixing, dialysised v-SNARE liposomes were mixed with dialysised t-SNAREs liposomes in the ratio of 1:1 and 4.5 μM concentration. For the fusion reaction performed with Munc18, at the beginning, different ratios of t-SNAREs liposomes and Munc18 were incubated at 4°C for about 2-3 h. Then the mixture was mixed with v-SNARE liposomes again to perform in vitro liposome fusion assay. The final reaction volume for each assay was 100 ul with total 1 mM lipids in Hepes buffer. Fluorescence intensity was monitored with the excitation and emission wavelengths of 465 and 530 nm, respectively. The fluorescence signal was recorded by a Varian Cary Eclipse model fluorescence spectrophotometer using a quartz cell of 100 ul with a 2-mm path length. All of the lipids mixing experiments were performed at 35°C.

### Fusion data analysis

Based on previously work, fusion levels were transformed to fusion round [26,41,42].

### Network modeling, bifurcation and robustness analysis of parameters

To perform comparative network motif modeling, we develop a Cytoscape [21] plug-in software CytoModeler, which can easily perform network/motif construction, simulation and visualization in various ways and work with other sophisticated dynamical modeling software (detailed in Supplemental materials). It can be freely downloaded at http://vrac.iastate.edu/~jlv/cytomodeler/. The kinetics simulation and bifurcation analysis were completed in CytoModeler and Systems Biology Toolbox. Differential equations were solved using the ODE23s routine.

For robustness analysis of parameters, the work used the Latin Hypercube Sampling method. 2000 random parameter sets were generated with +/-30% variance relative to their original values (Additional file: Figure S3).

### Initial conditions, parameters and units

#### Initial conditions and units

The concentrations of reactant proteins are given in molar units. For the SNARE proteins such as SNAP25 and syntaxin, we followed the studies [43,44] which evaluated the concentration of these protein in a range of 0.1-100 μM. The essential regulatory proteins SM/Munc18 is expressed at much lower levels compared to SNARE proteins. In the simulation experiments, the initial concentrations of SNAREs are 4.5 μM and the initial concentrations of SM changed in range of 0 ~ 6 μM.

#### Rate constants

In our models, where available, we have relied on *in vivo *and *in vitro *biochemical experiments for parameter values [26,44-49]. In cases where the values of biochemical parameter were not known yet, we estimated physically reasonable values based on a previous modeling study [43] which provided invaluable information on mining biochemical experiments for parameter values *in vivo/in vitro *and also approaches to estimating unknown parameters. It should be stressed that these available rate constants are measured independently and under different secretion systems which may be different quantitatively. However, because the exocytosis process is highly conserved between different cell types, we integrated these rate constants into our kinetic equations which aim at providing insights into fundamental regulatory mechanisms of protein interaction among two essential protein families (SM and SNARE) during almost every type of exocytosis process [14,16]. Therefore our models can served as a framework for integration refinement from different systems through adding system-specific regulatory steps or fitting newly characterized kinetic features.

## Completing financial interests

The authors declare that they have no competing interests.

## Authors' contributions

TX, JST, XG, and JAD conceived the study. TX, XG and JAD coordinated the project and the manuscript preparation. JST and SSR carried out lipid mixing assay. TX carried out the data analysis and drafted the manuscript. All authors read and approved the final manuscript.

## Supplementary Material

Additional file 1 **Supplements for yeast and neuronal SM-SNARE network modeling**. The file includes: System reactions, equations and parameters used in the models for the *in silico *experiments and parameter robustness analysis [21,26,40,47,48,50-56]. Click here for file
