# Supplementary material for: Network motif comparison rationalizes Sec1/Munc18-SNARE regulation mechanism in exocytosis
Source: BMC Syst Biol. 2012 Mar 16;6:19. doi: 10.1186/1752-0509-6-19 (PMC3439672; doi:10.1186/1752-0509-6-19)
Supplement: Additional file 1 — Supplements for yeast and neuronal SM-SNARE network modeling. The file includes: System reactions, equations and parameters used in the models for the in silico experiments and parameter robustness analysis [21,26,40,47,48,50-56]. [file 1752-0509-6-19-S1.PDF]

Supplements for Comparative network motif design rationalizes Sec1/Munc18-SNARE regulation mechanism in exocytosis

	Syntaxin	SNAP25	Synaptobrevin	SM	t-SNARE complex	t-SNARE/SM complex	SNAREpin	SNAREpin/SM complex
**Yeast**	**ySyx**<br>Sso1p	**yS25**<br>Sec9p	**ySyb**<br>Snc1/2p	**ySM**<br>Sec1p	**ytSN**	**ytSNSM**	**ySN**	**ySNSM**
**Neuron**	**nSyx**<br>syntaxin-1	**nS25**<br>SNAP25	**nSyb**<br>VAMP2	**nSM**<br>Munc18-1	**ntSN**	**ntSNSM**	**nSN**	**nSNSM**

Table I Abbreviations of yeast and neuronal SM-SNARE systems

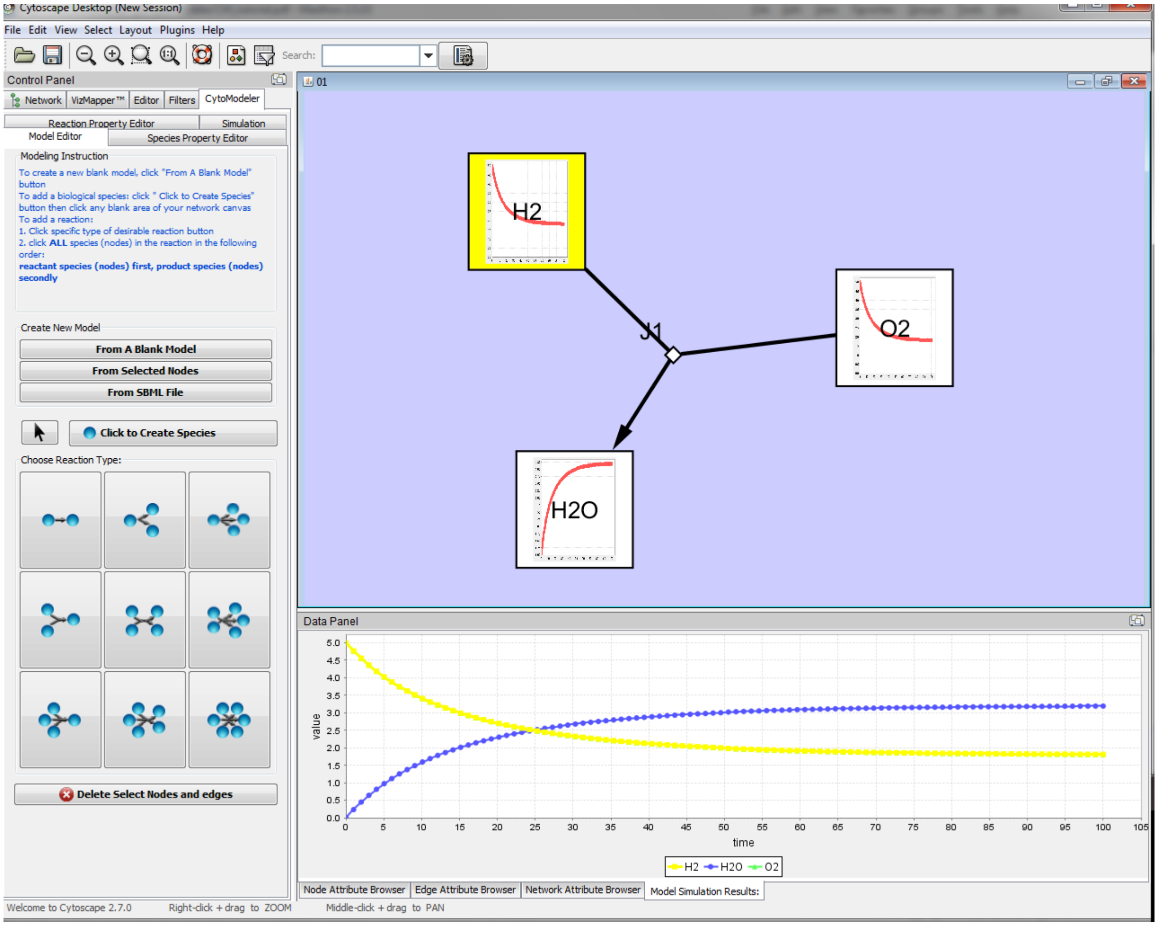

Figure S1 Modeling tool. To facilitate network motif model, we develop a software CytoModeler which is based on Cytoscape platform[1]. This tool provides: (i) graphical network editor for easy designing network motif model, (ii) compact built-in deterministic and stochastic simulators for easy simulation of models, (iii) model transformer for importing and exporting built network motif models in standard SBML format, (iv) static and dynamic/animated visualization of simulation results in network motif context, (v) model exchanger for co-working with the sophisticated modeling tools such as Systems Biology Toolbox2[2] and COPASI[3] to provide further analysis. Figure 1 shows an interface of CytoModeler.

Yeast SSN Reactions	Neuronal SSN Reactions
$ySyx + yS25 \underset{k_{-1}}{\overset{k_1}{\rightleftarrows}} ytSN$	$nSyx + nS25 \underset{k_{-1}}{\overset{k_1}{\rightleftarrows}} ntSN$
$ytSN + ySyb \underset{k_{-2}}{\overset{k_2}{\rightleftarrows}} ySN$	$ntSN + nSyb \underset{k_{-2}}{\overset{k_2}{\rightleftarrows}} nSN$
$ySN + ySM \underset{k_{-4}}{\overset{k_4}{\rightleftarrows}} ySNSM$	$nSyx + nSM \underset{k_{-3}}{\overset{k_3}{\rightleftarrows}} nSyxSMClosed$
$ytSN + ySM \underset{k_{-5}}{\overset{k_5}{\rightleftarrows}} ytSNSM$	$nSN + nSM \underset{k_{-4}}{\overset{k_4}{\rightleftarrows}} nSNSM$
$ytSNSM + ySyb \underset{k_{-6}}{\overset{k_6}{\rightleftarrows}} ySNSM$	$ntSN + nSM \underset{k_{-5}}{\overset{k_5}{\rightleftarrows}} ntSNSM$
$ySNSM \underset{k_{-f}}{\overset{k_{fsnsm}}{\rightleftarrows}} fusion_1$	$ntSNSM + nSyb \underset{k_{-6}}{\overset{k_6}{\rightleftarrows}} nSNSM$
$ySN \underset{k_{-f}}{\overset{k_{fsn}}{\rightleftarrows}} fusion_2$	$nSNSM \underset{k_{-f}}{\overset{k_{fsnsm}}{\rightleftarrows}} fusion_1$
$fusion = fusion_2 + fusion_1$	$nSN \underset{k_{-f}}{\overset{k_{fsn}}{\rightleftarrows}} fusion_2$
	$fusion = fusion_2 + fusion_1$

Table II The yeast and neuronal SM-SNARE systems

Yeast SSN Equation	Neuronal SSN Equation
$\frac{d(yS25)}{dt}=-v_1$<br>$\frac{d(ySyx)}{dt}=-v_1$<br>$\frac{d(ySyb)}{dt}=-v_2-v_6$<br>$\frac{d(ySM)}{dt}=-v_4-v_5$<br>$\frac{d(ytSN)}{dt}=v_1-v_2-v_5$<br>$\frac{d(ytSNSM)}{dt}=v_5-v_6$<br>$\frac{d(ySN)}{dt}=v_2-v_4-v_{fsn}$<br>$\frac{d(ySNSM)}{dt}=v_6+v_4-v_{fsnsm}$<br>$\frac{d(fusion)}{dt}=v_{fsn}+v_{fsnsm}$<br><br>$v_1=k_1\cdot ySyx\cdot yS25-k_{-1}ytSN$<br>$v_2=k_2\cdot ytSN\cdot ySyb-k_{-2}\cdot ySN$<br>$v_4=k_4\cdot ySN\cdot ySM-k_{-4}\cdot ySNSM$<br>$v_5=k_5\cdot ytSN\cdot ySM-k_{-5}\cdot ytSNSM$<br>$v_6=k_6\cdot ytSNSM\cdot ySyb-k_{-6}\cdot ySNSM$<br>$v_{fsn}=k_{fsn}\cdot ySN-k_{-f}\cdot fusion_2$<br>$v_{fsnsm}=k_{fsnsm}\cdot ySNSM-k_{-f}\cdot fusion_1$	$\frac{d(nS25)}{dt}=-v_1$<br>$\frac{d(nSyx)}{dt}=-v_1-v_3$<br>$\frac{d(nSyb)}{dt}=-v_2-v_6$<br>$\frac{d(nSM)}{dt}=-v_4-v_5-v_3$<br>$\frac{d(ntSN)}{dt}=v_1-v_2-v_5$<br>$\frac{d(ntSNSM)}{dt}=v_5-v_6$<br>$\frac{d(nSN)}{dt}=v_2-v_4-v_{fsn}$<br>$\frac{d(nSNSM)}{dt}=v_6+v_4-v_{fsnsm}$<br>$\frac{d(nSyxSMClosed)}{dt}=v_3$<br>$\frac{d(fusion)}{dt}=v_{fsn}+v_{fsnsm}$<br><br>$v_1=k_1\cdot nSyx\cdot nS25-k_{-1}ntSN$<br>$v_2=k_2\cdot ntSN\cdot nSyb-k_{-2}\cdot nSN$<br>$v_3=k_3\cdot nSyx\cdot nSM$<br>$-k_{-3}\cdot nSyxSMClosed$<br>$v_4=k_4\cdot nSN\cdot nSM-k_{-4}\cdot nSNSM$<br>$v_5=k_5\cdot ntSN\cdot nSM-k_{-5}\cdot ntSNSM$<br>$v_6=k_6\cdot ntSNSM\cdot nSyb-k_{-6}\cdot nSNSM$<br>$v_{fsn}=k_{fsn}\cdot nSN-k_{-f}\cdot fusion_2$<br>$v_{fsnsm}=k_{fsnsm}\cdot nSNSM-k_{-f}\cdot fusion_1$

Table III The equation sets of modeling yeast and neuronal SM-SNARE systems, For the two SM-SNARE networks, we constructed dynamical models for them. The models consist of differential equations with a set of parameters and non-zero initial conditions, based on previously characterized interaction diagrams. For each model, the state of the system is described by the concentrations of all relevant protein $(C_1(t), C_2(t)..., C_n(t))$. The rates of reactions are dependent on these concentrations and on biochemical rate constant parameters $(k_1, k_2, ..., k_n)$. To describe the temporal behavior, systems of ODEs are provided in general form: $\frac{dC_i}{dt}=f_i(\boldsymbol{C},\boldsymbol{K})$ which describes the

$i=1,...,n$

evolution of a set of state variables $\boldsymbol{C} = C_1(t), C_2(t)..., C_n(t)$; $\boldsymbol{K}$ represent vectors of system parameters. It is assumed that the state C evolves in a subset $\boldsymbol{C}$ of Euclidean space, in which it is positive or non-negative that is always satisfied in biochemical application. In our model, we used protein family names: syntaxin, SNAP25, VAMP and SM to present model variable names for specific SNARE proteins and SM protein in different organisms, respectively.

Parameter	Value	Reference
k_1	$1.6\times10^4 M^{-1}s^{-1} \sim 7\times10^4 M^{-1}s^{-1}$	[4-7]
k_{-1}	$5\times10^{-3} s^{-1} \sim 7\times10^{-2} s^{-1}$	[4-6]
k_2	$4\times10^6 M^{-1}s^{-1} \sim 6\times10^6 M^{-1}s^{-1}$	[4-6]
k_{-2}	$2\times10^{-4} s^{-1} \sim 7\times10^{-4} s^{-1}$	[4-6]
k_3	$6\times10^6 M^{-1}s^{-1} \sim 7\times10^6 M^{-1}s^{-1}$	[8]
k_{-3}	$9\times10^{-4} s^{-1} \sim 7\times10^{-3} s^{-1}$	[8, 9]
k_4	$6\times10^6 M^{-1}s^{-1} \sim 9\times10^6 M^{-1}s^{-1}$	[8, 10]
k_{-4}	$6\times10^{-5} s^{-1} \sim 5\times10^{-4} s^{-1}$	[8, 10]
k_5	$3\times10^6 M^{-1}s^{-1} \sim 7\times10^6 M^{-1}s^{-1}$	Est.
k_{-5}	$5\times10^{-4} s^{-1} \sim 7\times10^{-3} s^{-1}$	Est.
k_6	$3\times10^6 M^{-1}s^{-1} \sim 7\times10^6 M^{-1}s^{-1}$	Est.
k_{-6}	$5\times10^{-4} s^{-1} \sim 7\times10^{-3} s^{-1}$	Est.
k_{fsn}	$1.5\times10^{-3} s^{-1} \sim 5\times10^{-3} s^{-1}$	[11, 12]
k_{fsnsm}	$1.5\times10^{-2} s^{-1} \sim 1\times10^{-1} s^{-1}$	[10, 12]
k_{-f}	$1.\times10^{-3} s^{-1} \sim 8\times10^{-3} s^{-1}$	[11]

Table IV System parameters of the models. Est. Estimated; Units: The total amounts of different species are expressed in units of molar (M). The first and second rate constants presented in units of s^{-1} and $M^{-1}\cdot s^{-1}$, respectively.

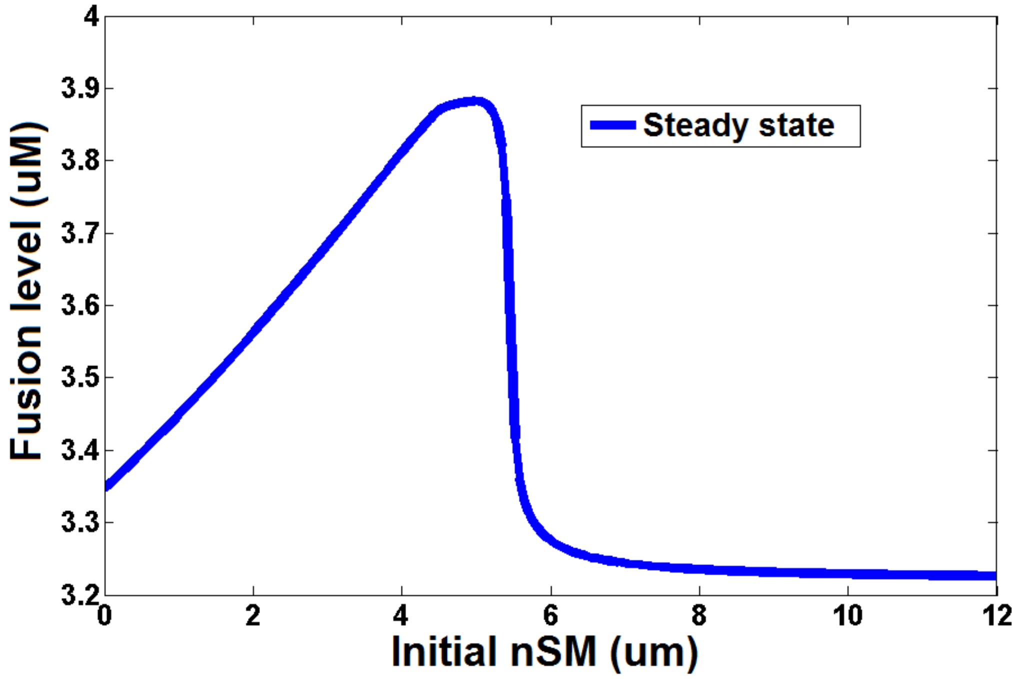

Figure S2 Bifurcation behavior of neuronal SM-SNARE system with displacement factor

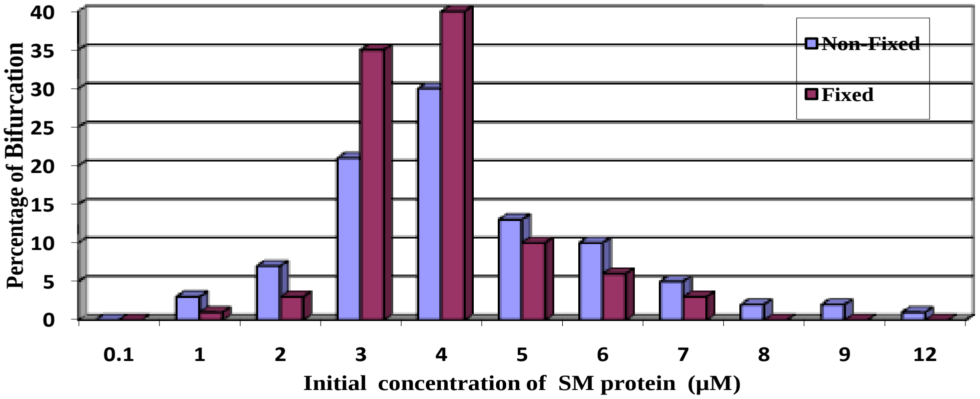

Figure S3 Robustness of bifurcation in feedback neuronal SM-SNARE system. For any given initial concentration of SM protein (0.1μM, 12 μM), the percentage of parameter sets that exhibits bifurcation are plotted. No Fix: All of the parameters are varied +/-30% from its default value. Fix: The key reaction constant rates of forming SNAREpin (k_1,k_{-1},k_2, *and* k_{-2}) are fixed while all other parameters are varied +/-30% from its default value for each sample run.

References

1. Shannon, P., et al., *Cytoscape: a software environment for integrated models of biomolecular interaction networks.* Genome Res, 2003. **13**(11): p. 2498-504.
2. Schmidt, H. and M. Jirstrand, *Systems Biology Toolbox for MATLAB: a computational platform for research in systems biology.* Bioinformatics, 2006. **22**(4): p. 514-5.
3. Hoops, S., et al., *COPASI--a COmplex PAthway SImulator.* Bioinformatics, 2006. **22**(24): p. 3067-74.
4. Nicholson, K.L., et al., *Regulation of SNARE complex assembly by an N-terminal domain of the t-SNARE Sso1p.* Nat Struct Biol, 1998. **5**(9): p. 793-802.
5. Pobbati, A.V., A. Stein, and D. Fasshauer, *N- to C-terminal SNARE complex assembly promotes rapid membrane fusion.* Science, 2006. **313**(5787): p. 673-6.
6. Margittai, M., et al., *Single-molecule fluorescence resonance energy transfer reveals a dynamic equilibrium between closed and open conformations of syntaxin 1.* Proc Natl Acad Sci U S A, 2003. **100**(26): p. 15516-21.
7. Fasshauer, D. and M. Margittai, *A transient N-terminal interaction of SNAP-25 and syntaxin nucleates SNARE assembly.* J Biol Chem, 2004. **279**(9): p. 7613-21.
8. Burkhardt, P., et al., *Munc18a controls SNARE assembly through its interaction with the syntaxin N-peptide.* Embo J, 2008. **27**(7): p. 923-33.

9. Pevsner, J., et al., *Specificity and regulation of a synaptic vesicle docking complex.* Neuron, 1994. **13**(2): p. 353-61.
10. Shen, J., et al., *Selective activation of cognate SNAREpins by Sec1/Munc18 proteins.* Cell, 2007. **128**(1): p. 183-95.
11. Lu, X., et al., *Membrane fusion induced by neuronal SNAREs transits through hemifusion.* J Biol Chem, 2005. **280**(34): p. 30538-41.
12. Tareste, D., et al., *SNAREpin/Munc18 promotes adhesion and fusion of large vesicles to giant membranes.* Proc Natl Acad Sci U S A, 2008. **105**(7): p. 2380-5.
